# Trends in Up-To-Date Colorectal Cancer Screening Among U.S. Adults Aged 50–75 Years and Variations by Race/Ethnicity and U.S. Census Bureau Divisions

**DOI:** 10.1016/j.focus.2022.100055

**Published:** 2022-12-10

**Authors:** Itunu O. Sokale, Omar Rosales, Jane R. Montealegre, Abiodun O. Oluyomi, Aaron P. Thrift

**Affiliations:** 1 Section of Epidemiology and Population Sciences, Department of Medicine, Baylor College of Medicine, Houston, Texas; 2 Dan L. Duncan Comprehensive Cancer Center, Baylor College of Medicine, Houston, Texas; 3 Department of Pediatrics, Baylor College of Medicine, Houston, Texas

**Keywords:** Colorectal cancer screening, racial/ethnic groups, U.S. Census Bureau Divisions, geographic locations, screening disparities

## Abstract

**Introduction:**

Mortality rates from colorectal cancer have declined over the past decades owing to population-based life-saving screening interventions. However, screening inequalities continue among racial and ethnic minorities despite having a higher disease burden. In this study, we assessed the patterns of up-to-date colorectal cancer screening rates among racial/ethnic groups across the U.S. Census Bureau Divisions.

**Methods:**

This population-based cross-sectional study used weighted data from 4 cycles of the Behavioral Risk Factors Surveillance System (2014, 2016, 2018, and 2020) of adults aged 50‒75 years without a previous diagnosis of colorectal cancer. The primary outcome was guideline-recommended up-to-date colorectal cancer screening. We used logistic regression models to examine temporal trends in up-to-date colorectal cancer screening from 2014 to 2020. In addition, we conducted detailed descriptive statistics of up-to-date screening rates, comparing trends in 2020 with those in 2014 overall by race/ethnicity and U.S. census divisions.

**Results:**

The overall proportion of individuals with up-to-date colorectal cancer screening increased from 66.5% in 2014 to 72.5% in 2020 (*p*<0.001). For racial/ethnic subgroups, from 2014 to 2020, screening rates increased significantly among non-Hispanic Whites (68.5%‒74.5%, *p*<0.001), non-Hispanic Blacks (68.0%‒74.6%, *p*<0.001), and Hispanics (51.5%‒62.8%, *p*<0.001). However, increases were not observed in all U.S. Census Bureau Divisions.

**Conclusions:**

Although colorectal cancer screening rates improved over time, they fall short of the 80% target. Substantial racial/ethnic and geographic disparities remain. Future studies investigating the factors influencing these disparities are needed.

## HIGHLIGHTS

• Up-to-date colorectal cancer screening rates have improved generally.
• Screening rates remain below the national Colorectal Cancer Roundtable target of 80%.
• Considerable racial/ethnic and place-based disparities exist in guideline-consistent colorectal cancer screening.

## INTRODUCTION

Colorectal cancer (CRC) is the third leading cause of cancer-related deaths, claiming >50,000 lives each year in the U.S.^1^ An estimated 150,000 new cases of CRC are projected for the U.S. in 2022,^2^ and most cases occur in people aged over 50 years.^3^^,^^4^ Screening for CRC, detection, and removal of precancerous lesions offer the opportunity to reduce CRC incidence. Hence the U.S. Preventive Services Task Force (USPSTF) recommends regular CRC screening in all adults aged 50–75 years (A recommendation) and, in 2021, updated screening guidelines to include adults aged 45–49 years at average risk for CRC (B recommendation).^3^ In addition, the American Cancer Society recommends routine screening for adults with average risk starting from age 45 years.^5^^,^^6^ Multiple modalities for CRC screening are available, including an annual high-sensitivity fecal occult blood test every year or fecal immunochemical test (FIT), stool DNA-FIT every 1–3 years, computed tomography (CT) colonography or flexible sigmoidoscopy every 5 years, flexible sigmoidoscopy every 10 years plus annual FIT, or colonoscopy every 10 years.^3^

The U.S. Centers for Disease Control and Prevention *Healthy People 2030* and the National Colorectal Cancer Roundtable set targets of 74.4% and 80%, respectively, for the proportion of eligible adults with guideline-concordant CRC screening.^7^^,^^8^ However, overall screening rates remain below these targets.^9^, ^10^, ^11^, ^12^ CRC incidence and mortality rates are highest among racial and ethnic minorities, specifically among non-Hispanic Blacks (NHBs) and Alaska Natives.^4^^,^^13^ Contributing to the disproportionate burden among racial and ethnic minorities is the fact that minorities have the lowest CRC screening rates.^9^^,^^10^

In addition to racial and ethnic disparities in screening rates, CRC screening rates in the U.S. vary geographically.^12^^,^^14^ In their population-based study using 2012 and 2016 data from the Behavioral Risk Factors Surveillance System (BRFSS), Joseph and colleagues^14^ reported an upward trend in up-to-date CRC screening in most of the U.S. However, rates declined in a few states, with Georgia having the greatest decrease (–4.4%).^14^ In addition, Richardson et al.^12^ found decreasing rates of the proportions of eligible U.S. adults who have never been screened for CRC from 2012 to 2020. Improvements varied by state, ranging from 1.2 percentage points in New Hampshire to 13.5 percentage points in South Dakota.^12^ Nevertheless, these studies did not disaggregate by race/ethnicity, potentially masking regional disparities in up-to-date trends.

Although overall screening rates may be increasing, the gains in screening coverage may not be similar across the board, particularly among racial and ethnic minorities. Previous studies have examined CRC screening trends by race/ethnicity^2^ and by state^14^; nonetheless, studies providing comprehensive data on up-to-date CRC screening rate patterns in different geographic areas in the U.S. by race/ethnicity are scarce. Thus, this study builds on previous scientific work and fills a critical gap in knowledge by describing recent temporal trends in up-to-date CRC screening overall, by race/ethnicity, and relative changes over time across multiple racial/ethnic groups in the 9 U.S. Census Bureau divisions^15^ from 2014 to 2020. Division-level data offer the opportunity to examine larger units of populations with similar demographics, cultural norms, and economies.^16^

## METHODS

### Study Population

This population-based cross-sectional study used data from 4 cycles of the BRFSS (2014, 2016, 2018, and 2020), a national state-based telephone-based survey of the non-institutionalized U.S. population aged ≥18 years. BRFSS collects data annually on health risk behaviors, healthcare access, preventive service utilization, and chronic health conditions. To ensure that the sampled population is representative of the U.S. population, BRFSS uses complex survey sampling and weighting methods. The BRFSS sampling method, data quality, and weighting methods have been published elsewhere.^17^

BRFSS collects CRC screening‒related data on even-numbered years, and our study sample included respondents aged 50–75 years from 2014, 2016, 2018, and 2020 surveys. On the basis of USPSTF CRC screening recommendations at the time of the BRFSS surveys included in this study, we restricted our study population to individuals aged 50–75 years. In addition, we excluded respondents with a previous diagnosis of CRC and those from the U.S. territories (Guam, Puerto Rico, or the U.S. Virgin Islands) owing to differences in healthcare delivery and small sizes of some racial/ethnic groups. Appendix Figure 1 (available online) shows the study sample selection, with inclusion and exclusion criteria. The final sample for this study was 779,143, including 442,935 females and 336,208 males. This study did not require IRB approval because it analyzed publicly available, deidentified data.

### Measures

The outcome variable was up-to-date CRC screening, that is, respondents aged 50–75 years who fully met the USPSTF CRC screening recommendations for each survey year. This was ascertained in the BRFSS on the basis of answers to questions about which of the recommended CRC tests a respondent completed, if any, and when they had the most recent test. Depending on the CRC screening guidelines when these answers were provided, the BRFSS computed a variable for up-to-date CRC screening. Of note, the USPSTF-recommended CRC tests included stool blood test within the past year or 3 years, colonoscopy within the past 10 years, sigmoidoscopy within the past 5 years, or blood stool test within the past 3 years for survey years 2014–2018. On the basis of emerging scientific evidence supporting the effectiveness of newer methods and the 2018 updated USPSTF guidelines, the 2020 survey was expanded to include stool DNA tests in the past 3 years and CT colonography in the past 5 years.^3^

**Covariates**. Race/ethnicity was categorized as non-Hispanic White (NHW), NHB, Hispanic, American Indian or Alaskan Native (AI/AN), non-Hispanic Asian and Pacific Islander or Native Hawaiian (API), and individuals of non-Hispanic other races and multiracial individuals (NHO). Geographic divisions were classified according to the 9 U.S. Census Bureau Divisions using the state Federal Information Processing Standard codes provided in the BRFSS data. The U.S. has different geographic levels, including the U.S. Census Bureau regions and divisions. Each of the 4 U.S. Census Bureau regions (Northeast, Midwest, South, and West) was subdivided into 2 or 3 divisions, making 9 divisions in total, on the basis of combinations of several states, including the District of Columbia.^15^^,^^16^ The divisions represent large geographic areas with similar historical development, demographic characteristics, and economies.^16^ See Appendix Table 1 (available online) for U.S. Census Bureau regions and divisions with states. As mentioned, survey years were 2014, 2016, 2018, and 2020. Covariates for the analysis included the following: age (50–59, 60–69, 70–75 years), sex (male and female), education (less than high school, high school, and higher than high school), annual household income (<$25,000, $25,000–$74,999, $75,000 or more, and missing [missing >13%]), and health insurance coverage (yes/no).

### Statistical Analysis

We estimated annual rates of up-to-date CRC screening among eligible individuals overall, by survey years, and by racial/ethnic groups. Overall and race-specific trends in up-to-date CRC screening were conducted from 2014 to 2020. Unadjusted trends as well as trends adjusted for age and sex (including race/ethnicity for models for overall participants) were conducted using logistic regression models with survey year treated as a continuous variable. Analysis was conducted using Stata, Version 17.0 (College Station, TX). In addition, we assessed the proportions of participants who were up to date with their CRC tests overall, by racial/ethnic groups across the U.S. Census Divisions in 2014 and 2020. Finally, we determined the relative change in up-to-date CRC screening in 2020 versus in 2014, overall and by racial/ethnic groups for each division. Results of the absolute and relative changes in CRC screening across the U.S. Census Divisions were displayed using a series of maps. ArcGIS Pro, Version 2.8 (Esri, Redlands, CA), was used to create these maps. We interpreted statistical significance as a 2-sided *p*<0.05. We excluded respondents who refused to answer, who were not sure, or who reported *don't know* to variables of interest, except for annual household income.

## RESULTS

Sample characteristics are presented in detail in Table 1. Briefly, >70% of participants were NHW, slightly over half were female, and about 55% were aged ≥60 years. Hispanics had the greatest proportion of less than high school–educated respondents (43.8%) and annual household income <$25,000 (40.4%) compared with other racial/ethnic groups. Conversely, APIs had the highest proportion of greater than high school educational attainment (77.1%) and an annual income of $75,000 or more (40.5%). See Appendix Table 2 (available online) for sample characteristics by survey year.

**Table 1:** Sample Characteristics by Overall and by Race/Ethnicity, Behavioral Risk Factors Surveillance System 2014–2020

Characteristics	Overall (N=779,143)	Non-Hispanic White (*n*=645,928)	Non-Hispanic Black (*n*=58,534)	Hispanic (*n*=34,216)	Non-Hispanic AI/AN (*n*=11,881)	Non-Hispanic API (*n*=11,479)	Non-Hispanic other (*n*=17,105)
	Sample *n* (weighted %)	Sample *n* (weighted %)	Sample *n* (weighted %)	Sample *n* (weighted %)	Sample *n* (weighted %)	Sample *n* (weighted %)	Sample *n* (weighted %)
Sex							
Male	336,208 (47.6)	280,443 (47.5)	21,836 (46.1)	14,938 (48.8)	5,162 (49.2)	5,661 (50.7)	8,168 (49.0)
Female	442,935 (52.4)	365,485 (52.5)	36,698 (53.9)	19,278 (51.2)	6,719 (50.8)	5,818 (49.3)	8,937 (51.0)
Age, years							
50–59	280,794 (44.5)	223,209 (42.1)	23,689 (49.0)	17,057 (54.8)	5,188 (50.8)	4,972 (49.0)	6,679 (46.4)
60–69	335,905 (39.1)	281,682 (40.1)	24,991 (37.8)	12,537 (34.0)	4,840 (36.1)	4,562 (38.3)	7,293 (38.8)
70–75	162,444 (16.4)	141,037 (17.8)	9,854 (13.1)	4,622 (11.2)	1,853 (13.1)	1,945 (12.7)	3,133 (14.8)
Education							
<High school	48,882 (12.5)	28,260 (7.5)	7,129 (17.1)	9,988 (43.8)	1,736 (22.2)	446 (6.9)	1,323 (11.7)
High school	211,068 (27.6)	172,929 (28.7)	19,236 (30.2)	8,687 (21.3)	3,738 (31.0)	2,006 (16.0)	4,472 (24.5)
>High school	519,193 (59.9)	444,739 (63.8)	32,169 (52.7)	15,541 (34.9)	6,407 (46.8)	9,027 (77.1)	11,310 (63.8)
Household income							
<$25,000	160,781 (21.3)	114,075 (16.5)	21,442 (34.7)	13,105 (40.4)	5,140 (40.0)	1,909 (15.9)	5,110 (27.6)
$25,000–74,999	281,119 (34.0)	238,694 (35.1)	19,124 (32.9)	10,217 (29.8)	3,643 (30.0)	3,778 (29.8)	5,663 (31.1)
$75,000 or more	233,016 (31.3)	207,528 (35.3)	9,764 (19.0)	5,815 (14.9)	1,598 (17.0)	4,380 (40.5)	3,931 (26.1)
Missing	104,227 (13.4)	85,631 (13.1)	8,204 (13.4)	5,079 (14.9)	1,500 (13.0)	1,412 (13.8)	2,401 (15.2)
Health insurance							
Yes	738,835 (92.8)	618,235 (94.9)	54,007 (90.7)	28,683 (80.5)	11,057 (90.9)	10,874 (93.8)	15,979 (91.9)
No	40,308 (7.2)	27,693 (5.1)	4,527 (9.3)	5,533 (19.5)	824 (9.1)	605 (6.2)	1,126 (8.1)
Up-to-date CRC screening							
Yes	560,738 (68.9)	471,523 (71.1)	42,543 (69.4)	20,316 (55.3)	7,078 (59.8)	7,706 (63.6)	11,572 (65.0)
No	218,405 (31.1)	174,405 (28.9)	15,991 (30.6)	13,900 (44.7)	4,803 (40.1)	3,773 (36.4)	5,533 (35.0)
U.S. Census Bureau divisions^a^							
I. New England	92,569 (5.1)	84,746 (6.0)	2,141 (2.0)	2,727 (2.8)	603 (3.2)	656 (3.1)	1,696 (5.4)
2. Middle Atlantic	66,153 (13.0)	55,259 (13.0)	4,798 (13.4)	3,525 (12.6)	424 (6.2)	1,007 (17.6)	1,140 (9.8)
3. East North Central	75,790 (15.3)	66,546 (17.3)	5,420 (14.5)	1,356 (6.1)	700 (8.9)	472 (5.8)	1,296 (10.3)
4. West North Central	131,270 (6.9)	120,758 (8.5)	3,330 (3.1)	2,404 (1.6)	2,640 (8.8)	562 (1.9)	1,576 (5.3)
5. South Atlantic	146,537 (21.2)	110,958 (20.2)	25,110 (35.3)	4,917 (17.0)	1,321 (17.9)	1,324 (11.3)	2,907 (19.1)
6. East South Central	47,016 (6.0)	35,984 (6.5)	9,350 (9.3)	285 (0.6)	428 (6.4)	143 (0.6)	826 (4.7)
7. West South Central	49,541 (11.0)	36,474 (9.4)	5,773 (13.6)	4,537 (21.5)	1,063 (14.9)	320 (4.9)	1,374 (10.1)
8. Mountain	105,978 (7.1)	88,562 (7.3)	1,369 (2.0)	9,752 (10.7)	3,408 (17.4)	853 (4.7)	2,034 (7.3)
9. Pacific	64,289 (14.4)	46,641 (11.9)	1,243 (6.8)	4,713 (27.1)	1,294 (16.3)	6,142 (50.1)	4,256 (27.9)

a U.S. Census Bureau Divisions with states in each division: Division 1: New England (Connecticut, Maine, Massachusetts, New Hampshire, Rhode Island, Vermont), Division 2: Middle Atlantic (New Jersey, New York, Pennsylvania), Division 3: East North Central (Indiana, Illinois, Michigan, Ohio, Wisconsin), Division 4: West North Central (Iowa, Kansas, Minnesota, Missouri, Nebraska, North Dakota, South Dakota), Division 5: South Atlantic (Delaware, District of Columbia, Florida, Georgia, Maryland, North Carolina, South Carolina, Virginia, West Virginia), Division 6: East South Central (Alabama, Kentucky, Mississippi, Tennessee), Division 7: West South Central (Arkansas, Louisiana, Oklahoma, Texas), Division 8: Mountain (Arizona, Colorado, Idaho, New Mexico, Montana, Utah, Nevada, Wyoming), and Division 9: Pacific (Alaska, California, Hawaii, Oregon, Washington). AI/AN, American Indian/Alaskan Natives; API, non-Hispanic Asian and Pacific Islander or Native Hawaiian; CRC, colorectal cancer.

**Figure 1:**
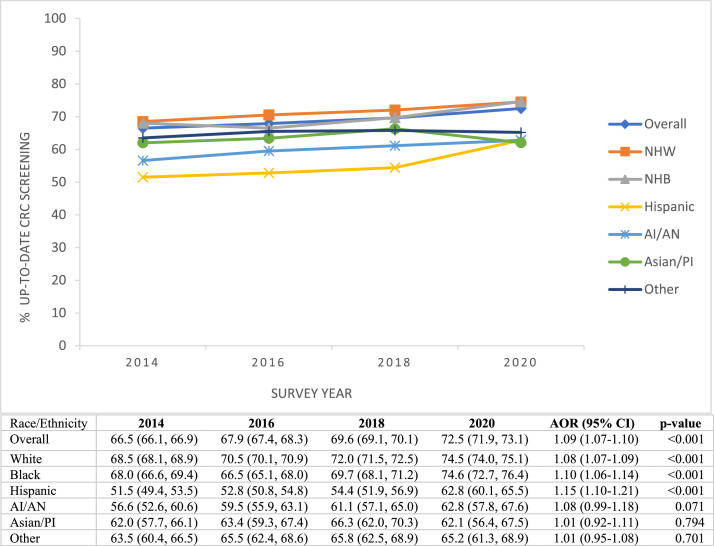
Up-to-date CRC screening from 2014 to 2020. This figure shows weighted proportions of participants who fully met USPSTF CRC screening recommendations overall and by racial/ethnic groups by survey year, Behavioral Risk Factors Surveillance System 2014–2020 (N=779,143). AOR and *p-*values are based on adjusted logistic regression models per year increase in the odds of having up-to-date CRC screening. The model for the overall population was adjusted for age, sex, and race/ethnicity, whereas racial/ethnic subgroup analysis controlled for age and sex. The results are weighted to reflect the U.S. population according to the complex survey design. AI/AN, American Indians and Alaskan Natives; Asian/PI, Asian/Pacific Islanders; CRC, colorectal cancer; NHB, non-Hispanic Black; NHW, non-Hispanic White; USPSTF, U.S. Preventive Services Task Force.

From 2014 to 2020, an estimated 68.9% of individuals of screening age in the U.S. were up to date with the USPSTF-recommended CRC screening. Figure 1 shows trends in CRC screening rates overall and by race/ethnicity. The proportion of individuals of screening age in the U.S. up to date with CRC screening increased from 66.5% in 2014 to 72.5% in 2020 (*p*<0.001). When examined by race/ethnicity, from 2014 to 2020, CRC screening rates increased significantly among NHWs (68.5%‒74.5%, *p*<0.001), NHBs (68.0%‒74.6%, *p*<0.001), and Hispanic (51.5%‒62.8%, *p*<0.001). Conversely, CRC screening rates remained stable among AI/ANs, APIs, and NHOs. Hispanics had the lowest screening rates in all survey years except in 2020 (62.8%), when rates among APIs dropped to 62.1%. Although gaps in screening between NHWs and Hispanics narrowed from 17% in 2014 to 11.7% in 2020 (absolute difference), large disparities remain between NHWs and APIs (12.4% difference) and between NHWs and Hispanics or AI/ANs (11.7% difference) in 2020.

**Figure 2:**
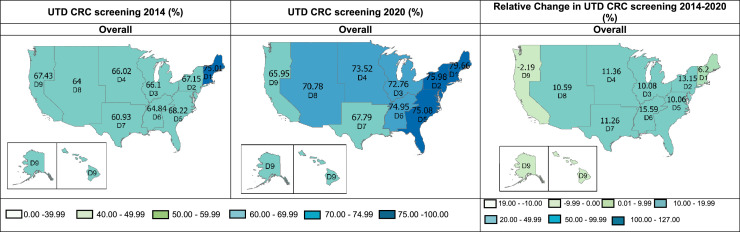
Up-to-date CRC screening across U.S. census divisions. This figure shows weighted UTD CRC screening among U.S. adults aged 50–75 years, overall, across U.S. Census Bureau Divisions, *Behavioral Risk Factors Surveillance System, 2014 and 2020. Percentages were weighted to reflect the U.S. population according to the complex survey design. The relative percentage change was calculated by dividing the difference in screening rates (2020 minus 2014) by the 2014 rate and then multiplying by 100. *p*-values from adjusted logistic regression models of screening rates in 2020 versus in 2014 by division: Divisions 1–8≤0.001; Division 9=0.274. Models adjusted for age, sex, and race/ethnicity. *U.S. Census Bureau Divisions with states in each division: Division 1: New England (Connecticut, Maine, Massachusetts, New Hampshire, Rhode Island, Vermont), Division 2: Middle Atlantic (New Jersey, New York, Pennsylvania), Division 3: East North Central (Indiana, Illinois, Michigan, Ohio, Wisconsin), Division 4: West North Central (Iowa, Kansas, Minnesota, Missouri, Nebraska, North Dakota, South Dakota), Division 5: South Atlantic (Delaware, District of Columbia, Florida, Georgia, Maryland, North Carolina, South Carolina, Virginia, West Virginia), Division 6: East South Central (Alabama, Kentucky, Mississippi, Tennessee), Division 7: West South Central (Arkansas, Louisiana, Oklahoma, Texas), Division 8: Mountain (Arizona, Colorado, Idaho, New Mexico, Montana, Utah, Nevada, Wyoming), and Division 9: Pacific (Alaska, California, Hawaii, Oregon, Washington). CRC, colorectal cancer; UTD, up-to-date.

In 2014, the overall proportion of eligible individuals in the U.S. with up-to-date CRC screening was below 70% in all divisions except in Division 1 (New England; 75.01%) (Figure 2). By 2020, the proportion of individuals of screening age in the U.S. up-to-date with CRC screening had increased nationwide (*p*≤0.001) except in Division 9 (Pacific), where there was a slight relative but not significant decrease in up-to-date CRC (–2.19%). In addition, 7 of 9 U.S. divisions had rates above 70% in 2020. Absolute increases in screening rates ranged from 6.2% to 15.59%. Appendix Tables 3 and 4 (available online) summarize the distribution of the study sample by the U.S. Census Divisions and up-to-date CRC screening rates for the 50 states and Washington, DC, respectively.

**Figure 3:**
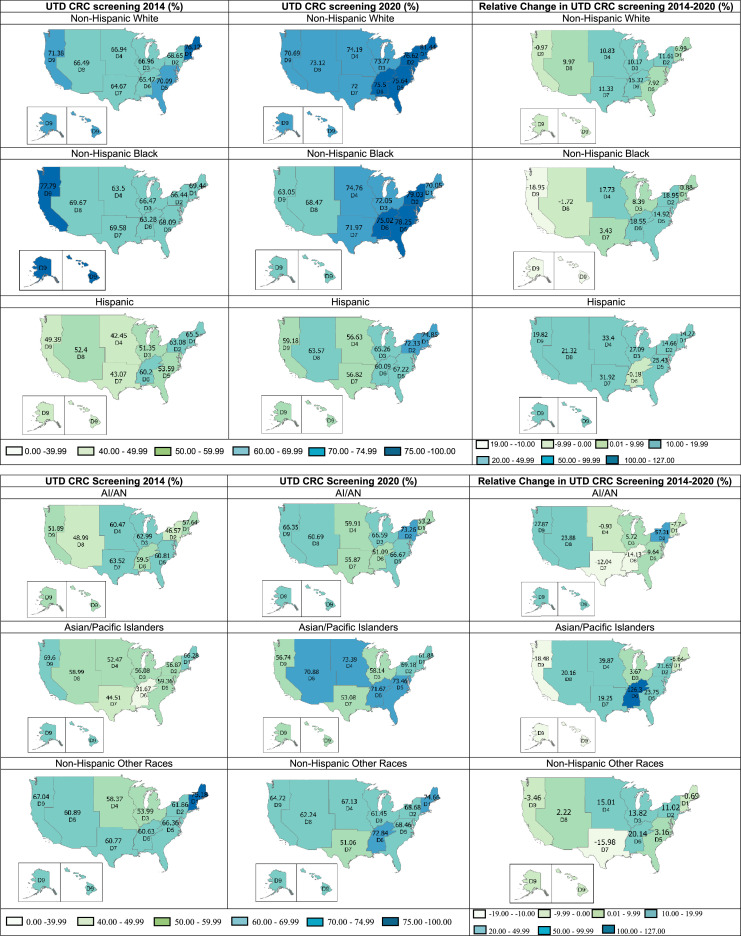
Up-to-date colorectal cancer screening by race/ethnicity across U.S. census divisions. This figure shows the weighted UTD CRC screening among U.S. adults aged 50–75 years, by race/ethnicity across U.S. Census Bureau Divisions, *Behavioral Risk Factors Surveillance System, 2014 and 2020. Percentages were weighted to reflect the U.S. population according to the complex survey design. The relative percentage change was calculated by dividing the difference in screening rates (2020 minus 2014) by the 2014 rate and then multiplying by 100. *p*-values from adjusted logistic regression models of screening rates in 2020 versus in 2014 are presented in Appendix Table 5 (available online). *U.S. Census Bureau Divisions with states in each division: Division 1: New England (Connecticut, Maine, Massachusetts, New Hampshire, Rhode Island, Vermont), Division 2: Middle Atlantic (New Jersey, New York, Pennsylvania), Division 3: East North Central (Indiana, Illinois, Michigan, Ohio, Wisconsin), Division 4: West North Central (Iowa, Kansas, Minnesota, Missouri, Nebraska, North Dakota, South Dakota), Division 5: South Atlantic (Delaware, District of Columbia, Florida, Georgia, Maryland, North Carolina, South Carolina, Virginia, West Virginia), Division 6: East South Central (Alabama, Kentucky, Mississippi, Tennessee), Division 7: West South Central (Arkansas, Louisiana, Oklahoma, Texas), Division 8: Mountain (Arizona, Colorado, Idaho, New Mexico, Montana, Utah, Nevada, Wyoming), and Division 9: Pacific (Alaska, California, Hawaii, Oregon, Washington). CRC, colorectal cancer; UTD, up-to-date.

A closer examination of screening rates by race/ethnicity (Figure 3 and Appendix Table 5, available online) showed variations across the U.S. Census Divisions. Up-to-date CRC screening rates among NHWs increased from 2014 to 2020 across all divisions (*p*<0.001) except across the Pacific (–0.97%, *p*=0.161). Despite that, screening rates among NHWs were above 70% in all divisions in 2020, with the highest rate of 81.44% in New England. Among NHBs, screening rates increased significantly in Divisions 2–6 and stabilized in other divisions except in the Mountain and Pacific, dropping from 69.67% to 68.47% (*p*=0.531) and from 77.79% to 63.05% in 2020, respectively (*p*=0.039). For Hispanics, CRC screening rates were relatively low, ranging from 42.45% to 65.50%, in all divisions in 2014. Although the proportion of Hispanics with up-to-date screening generally increased, the rates still fell behind other groups in 2020.

In 2014, the up-to-date CRC screening rates among AI/ANs ranged from 46.57% to 63.52% across census divisions (Figure 3). In addition, there were nonsignificant drops in screening rates in multiple divisions, including New England, West North Central, East South Central, and West South Central. However, Middle Atlantic had the greatest increase (57.31%, *p*=0.032) in the proportion of screening-compliant AI/ANs. For APIs, screening rates increased substantially in a few divisions. For instance, there was a 126.30% (*p*=0.003) increase in up-to-date screening in East South Central and 39.87% (*p*=0.015) in West North Central.

On the contrary, rates among APIs in the Pacific (*p*=0.034) declined. Finally, screening rates among NHOs stabilized in most places, yet only 2 divisions (New England and East South Central) had rates above 70% in 2020. The proportion of NHOs with up-to-date CRC screening reduced in New England, West South Central, and the Pacific, although not statistically significant. Finally, up-to-date CRC screening rates grew over time across most subgroups in Middle Atlantic and South Atlantic. Likewise, rates reduced substantially among NHBs and APIs in the Pacific. Appendix Table 5 (available online) shows the absolute and relative changes in overall screening rates and race/ethnicity.

## DISCUSSION

Our assessment of trends in CRC screening showed that the overall proportion of U.S. adults aged 50–75 years who were compliant with USPSTF guidelines increased significantly from 2014 to 2020, although improvement rates varied. These findings are consistent with those of previous studies that reported similar increases in CRC screening trends.^9^^,^^11^^,^^12^^,^^14^ A population-based study using the National Health Interview Survey data evaluated trends in CRC screening from 2008 to 2015. The study observed fluctuating trends in screening during the study period, with a steady increase from 2013 to 2015 (from 57.3% to 61.3%, *p*<0.05).^18^ Furthermore, we observed substantial racial/ethnic and geographic disparities in up-to-date CRC screening.

Although the rates among a few groups hit the *Healthy People 2030* target of 74.4%,^7^ they fell short of the NCCTR target of 80%^8^ in 2020. The overall increase in up-to-date CRC screening rates may be due to new health policies enhancing access to preventive services, including Medicaid expansion and state mandates of CRC screening coverage for eligible adults.^9^^,^^19^, ^20^, ^21^ Other possible explanations for the observed trends are expanding screening options with additional noninvasive and highly effective methods in the most recent years, such as stool DNA tests and CT colonography,^22^ and public health interventions to improve CRC screening uptake nationwide.^9^^,^^23^

Although guideline-consistent screening rates increased significantly among NHWs, NHBs, and Hispanics, screening rates were lowest overall among Hispanics, followed by the AI/ANs, throughout our study period. Screening rates among AI/ANs remained stable from 2014 to 2020, although they have the second-highest CRC mortality rate.^4^^,^^13^ Despite narrowing racial/ethnic CRC screening disparities over the years, wide gaps exist between NHWs and minority races except in NHBs, among whom the rates increased significantly. This may be due to increasing interventions specifically aimed to improve screening among NHBs, who have the greatest burden of CRC incidence and mortality compared with other racial/ethnic subgroups.^24^, ^25^, ^26^ Consistent with our findings, studies have documented racial and ethnic disparities in CRC screening.^9^^,^^27^ In their BRFSS-based study, May and colleagues^9^ reported racial/ethnic differences in CRC screening uptake trends from 2008 to 2016. The largest disparity was between NHWs (63.9% [in 2008] to 70.4% [in 2016]) and Hispanics (44.7% [in 2008] to 53.4% [in 2016]).

An examination of up-to-date CRC in 2014 versus in 2020 by geography for racial/ethnic groups combined revealed a slight decline in guideline-consistent screening in the Pacific census division. Declining screening rates in most groups except Hispanics and AI/ANs at the race/ethnicity-level analysis explains the falling rate overall in this division. Factors influencing the decline in up-to-date CRC screening rates across multiple groups in the Pacific are unclear. All states in this division expanded Medicaid in 2014, except Alaska, where expansion was implemented in 2015.^28^^,^^29^ We do not understand the mechanism underlying this division's decline in screening rates. Future studies could investigate multilevel barriers to screening in this area and other divisions with decreased rates, including knowledge, attitudes, culture, availability, and distance to screening facilities.

In addition, the within-division racial/ethnic disparities in CRC screening rates are noteworthy. For example, in West South Central, although the rates for NHWs and Hispanics increased relatively by 11.33% and 31.92%, respectively, the rates for AI/ANs and NHOs dropped by 12.04% and 15.98%, respectively. Again, although the rates among Hispanics and AI/ANs improved in the Pacific, the rates for other racial/ethnic subgroups declined. These findings highlight overlapping racial/ethnic and place-based inequalities in CRC screening in the U.S. Future studies should investigate the mechanisms and intersections of multilevel factors influencing these disparities.

### Limitations

Our study has several strengths, including providing recent data on up-to-date CRC screening trends among a nationally representative sample and being the first to report in-depth examinations of trends in guideline-consistent CRC screening by race/ethnicity across geographic locations in the U.S. In addition, our data provide crucial information on the progress with recommended screening nationwide among racial/ethnic groups. Notwithstanding, the results should be interpreted within the context of certain limitations. First, we used self-reported CRC screening variables, which may be subject to recall bias and social desirability bias. Again, BRFSS survey data do not provide information to elucidate the purpose of CRC tests. Thus, we are not able to differentiate whether CRC tests were conducted for surveillance or diagnostic purposes, and our screening prevalence data could include diagnostic tests. As mentioned earlier, the USPSTF and American Cancer Society recently dropped the routine screening commencement age to 45 years,^3^^,^^6^ but we could not examine trends in CRC screening rates among this group within our study period. Future studies might explore trends in the uptake of CRC screening in this population. Finally, we could not investigate racial/ethnic screening trends at the state level owing to the small sample sizes of some minority population subgroups in some states.

## CONCLUSIONS

In conclusion, our population-based study of recent trends in CRC screening confirms a generalized increase in up-to-date screening among adults aged 50–75 years from 2014 to 2020. However, our findings provide critical granular details about substantial racial/ethnic and place-based disparities in guideline-consistent screening. Future studies investigating the factors underpinning these disparities could inform the development of targeted interventions to address the barriers to up-to-date CRC screening among multiple vulnerable populations in different parts of the U.S.

## Declarations of interest

None.

## Appendix Supplementary materials

Image, application 1

## Footnotes

Supplementary material associated with this article can be found in the online version at doi:10.1016/j.focus.2022.100055.
