# Supplementary material for: Trends in Up-To-Date Colorectal Cancer Screening Among U.S. Adults Aged 50–75 Years and Variations by Race/Ethnicity and U.S. Census Bureau Divisions
Source: AJPM Focus. 2022 Dec 10;2(1):100055. doi: 10.1016/j.focus.2022.100055 (PMC10546535; doi:10.1016/j.focus.2022.100055)
Supplement: Supplementary file 1 [file mmc1.docx]

**Final Sample for Analysis**

(n=779,143)

Participants in 2014, 2016, 2018, 2020 BRFSS

(n=1,740,462)

Missing outcomes (n=162,798) and selected covariates

(n=26,296)

Had previous colorectal cancer disease

(n=3,653)

Aged <50 or >75

(n=767,446)

Missing gender (n=1,126)

n=968,237

Participants aged 50-75 (n=971,890)

All participants (n=1,739,336)

**Appendix Figure 1.** Flowchart of the selection of study population. A total of 779,143 participants were included in the current study using data from the Behavioral Risk Factor Surveillance System (BRFSS) 2014, 2016, 2018, and 2020.

**Description:** Figure 1 shows the flowchart of the selection of study population. A total of 779,143 participants were included in the current study using data from the Behavioral Risk Factor Surveillance System (BRFSS) 2014, 2016, 2018, and 2020.

**Appendix Table 1.** U.S. Census Bureau Regions and Divisions with States

| **Regions** | **Divisions** | **States** |
| --- | --- | --- |
| Region 1: Northeast | Division 1:  New England | Connecticut, Maine, Massachusetts, New Hampshire, Rhode Island, Vermont |
|  | Division 2: Middle Atlantic | New Jersey, New York, Pennsylvania |
| Region 2: Midwest | Division 3: East North Central | Indiana, Illinois, Michigan, Ohio, Wisconsin |
|  | Division 4: West North Central | Iowa, Kansas, Minnesota, Missouri, Nebraska, North Dakota, South Dakota |
| Region 3: South | Division 5: South Atlantic | Delaware, District of Columbia, Florida, Georgia, Maryland, North Carolina, South Carolina, Virginia, West Virginia |
|  | Division 6: East South Central | Alabama, Kentucky, Mississippi, Tennessee |
|  | Division 7: West South Central | Arkansas, Louisiana, Oklahoma, Texas |
| Region 4: West | Division 8: Mountain | Arizona, Colorado, Idaho, New Mexico, Montana, Utah, Nevada, Wyoming |
|  | Division 9: Pacific | Alaska, California, Hawaii, Oregon, Washington |

**Appendix Table 2.** Sample characteristics by survey year, Behavioral Risk Factor Surveillance System (BRFSS) 2014-2020

| **Characteristics** | **Overall**  (n=779,143) | **BRFSS 2014**  (n=211,492) | **BRFSS 2016**  (n=216,613) | **BRFSS 2018**  (n=187,082) | **BRFSS 2020**  (n=163,956) |
| --- | --- | --- | --- | --- | --- |
|  | Sample No (Weighted %) | Sample No (Weighted %) | Sample No (Weighted %) | Sample No (Weighted %) | Sample No (Weighted %) |
| Race/ethnicity  NH White  NH Black  Hispanic  AI/AN  API  Other | 645,928 (73.1)  58,534 (11.0)  34,216 (10.2)  11,881 (0.9)  11,479 (3.4)  17,105 (1.5) | 177,398 (74.5)  15,719 (11.2)  8,563 (9.1)  2,921 (1.0)  2,663 (2.8)  4,228 (1.4) | 180,359 (73.5)  16,429 (10.8)  9,118 (9.9)  3,070 (0.9)  3,072 (3.5)  4,565 (1.4) | 153,864 (73.1)  14,441 (10.6)  8,345 (10.2)  3,150 (0.9)  2,924 (3.7)  4,358 (1.5) | 134,307 (70.7)  11,945 (11.4)  8,190 (11.7)  2,740 (1.0)  2,820 (3.5)  3,954 (1.6) |
| Sex  Male  Female | 336,208 (47.6)  442,935 (52.4) | 86,821 (47.4)  124,671 (52.6) | 92,371 (47.7)  124,242 (52.3) | 83,424 (48.0)  103,658 (52.0) | 73,592 (47.6)  90,364 (52.4) |
| Age  50-59  60-69  70-75 | 280,794 (44.5)  335,905 (39.1)  162,444 (16.4) | 81,039 (47.6)  90,142 (37.6)  40,311 (14.8) | 78,197 (44.9)  94,959 (39.5)  43,457 (15.6) | 65,428 (43.1)  80,554 (39.6)  41,100 (17.3) | 56,130 (41.7)  70,250 (40.0)  37,576 (18.4) |
| Education  < High school  High school  > High School | 48,882 (12.5)  211,068 (27.6)  519,193 (59.9) | 13,814 (12.8)  59,470 (28.9)  138,208 (58.3) | 13,920 (12.8)  60,218 (28.4)  142,475 (58.8) | 11,906 (12.3)  49,723 (26.8)  125,453 (60.9) | 9,242 (11.8)  41,657 (26.0)  113,057 (62.3) |
| Household Income  <$25,000  $25,000-74,999  $75,000 or more  Missing | 160,781 (21.3)  281,119 (34.0)  233,016 (31.3)  104,227 (13.4) | 46,785 (23.5)  80,934 (36.5)  58,746 (28.9)  25,027 (11.1) | 45,942 (21.7)  79,342 (34.9)  62,630 (30.3)  28,699 (13.1) | 38,176 (20.6)  65,654 (32.7)  57,772 (32.6)  25,480 (14.1) | 29,878 (18.9)  55,189 (31.3)  53,868 (34.0)  25,021 (15.8) |
| Health Insurance  Yes  No | 738,835 (92.8)  40,308 (7.2) | 199,999 (92.2)  11,493 (7.8) | 206,428 (93.4)  10,185 (6.6) | 177,285 (92.9)  9,797 (7.1) | 155,123 (92.9)  8,833 (7.1) |
| Up-to-date CRC Screening  Yes  No | 560,738 (68.9)  218,405 (31.1) | 147,233 (66.5)  64,259 (33.5) | 154,651 (67.9)  61,962 (32.1) | 135,395 (30.4)  51,687 (69.6) | 123,459 (72.5)  40,497 (27.5) |
| US Census Bureau Divisions*  I. New England  2. Middle Atlantic  3. East North Central  4. West North Central  5. South Atlantic  6. East South Central  7. West South Central  8. Mountain  9. Pacific | 92,569 (5.1)  66,153 (13.0)  75,790 (15.3)  131,270 (6.9)  146,537 (21.2)  47,016 (6.0)  49,541 (11.0)  105,978 (7.1)  64,289 (14.4) | 24,962 (5.1)  13,956 (13.7)  20,763 (16.0)  38,129 (7.0)  34,183 (21.1)  14,361 (6.0)  15,870 (11.0)  34,049 (7.0)  15,219 (13.1) | 23,639 (5.0)  21,668 (13.3)  20,942 (15.2)  31,495 (6.9)  47,309 (21.1)  13,317 (6.2)  12,907 (10.8)  27,462 (6.8)  17,874 (14.7) | 22,638 (5.1)  18,168 (12.3)  17,731 (15.1)  31,589 (7.0)  36,633 (21.3)  11,121(5.7)  10,920 (11.0)  21,304 (7.3)  16,978 (15.2) | 21,330 (5.1)  12,361 (12.7)  16,354 (14.7)  30,057 (6.9)  28,412 (21.2)  8,217 (5.8)  9,844 (11.2)  23,163 (7.3)  14,218 (14.9) |

**Abbreviations**: AI/AN, American Indian/Alaskan Natives; API, non-Hispanic Asian and Pacific Islander or Native Hawaiian

*US Census Bureau Divisions with states in each division: Division 1: New England (Connecticut, Maine, Massachusetts, New Hampshire, Rhode Island, Vermont), Division 2: Middle Atlantic (New Jersey, New York, Pennsylvania), Division 3: East North Central (Indiana, Illinois, Michigan, Ohio, Wisconsin), Division 4: West North Central (Iowa, Kansas, Minnesota, Missouri, Nebraska, North Dakota, South Dakota), Division 5: South Atlantic (Delaware, District of Columbia, Florida, Georgia, Maryland, North Carolina, South Carolina, Virginia, West Virginia), Division 6: East South Central (Alabama, Kentucky, Mississippi, Tennessee), Division 7: West South central (Arkansas, Louisiana, Oklahoma, Texas), Division 8: Mountain (Arizona, Colorado, Idaho, New Mexico, Montana, Utah, Nevada, Wyoming), and Division 9: Pacific (Alaska, California, Hawaii, Oregon, Washington).

**Appendix Table 3.** Distribution of respondents aged 50-75 years, overall and by race/ethnicity across US Census Bureau Divisions, Behavioral Risk Factor Surveillance System 2014 and 2020

| **Survey Year** | **US Census Bureau Divisions*** | **Overall** | **Non-Hispanic White** | **Non-Hispanic Black** | **Hispanic** | **Non-Hispanic AI/AN** | **Non-Hispanic API** | **Non-Hispanic Other** |
| --- | --- | --- | --- | --- | --- | --- | --- | --- |
|  |  | Sample No (Weighted %) | Sample No (Weighted %) | Sample No (Weighted %) | Sample No (Weighted %) | Sample No (Weighted %) | Sample No (Weighted %) | Sample No (Weighted %) |
| 2014 | I. New England | 24,962 (5.1) | 22,948 (86.9) | 627 (4.1) | 672 (5.2) | 156 (0.6) | 180 (1.6) | 359 (1.6) |
|  | 2. Middle Atlantic | 13,956 (13.7) | 11,352 (73.3) | 1,372 (11.4) | 800 (9.4) | 68 (0.5) | 252 (4.7) | 112 (0.7) |
|  | 3. East North Central | 20,763 (16.0) | 18,499 (83.5) | 1,360 (10.5) | 286 (3.5) | 172 (0.5) | 120 (1.0) | 326 (1.0) |
|  | 4. West North Central | 38,129 (7.0) | 35,335 (90.3) | 1,013 (4.7) | 573 (1.9) | 642 (1.1) | 143 (0.7) | 423 (1.2) |
|  | 5. South Atlantic | 34,183 (21.1) | 25,847 (70.2) | 6,214 (18.8) | 883 (7.3) | 246 (0.9) | 303 (1.5) | 690 (1.3) |
|  | 6. East South Central | 14,361 (6.0) | 11,320 (80.0) | 2,590 (16.5) | 72 (0.9) | 129 (0.9) | 33 (0.3) | 217 (1.3) |
|  | 7. West South Central | 15,870 (11.0) | 11,729 (63.9) | 1,816 (13.4) | 1,467 (18.5) | 330 (1.3) | 117 (1.7) | 411 (1.2) |
|  | 8. Mountain | 34,049 (7.0) | 29,010 (77.8) | 429 (2.7) | 2,901 (13.7) | 876 (2.3) | 262 (2.2) | 571 (1.3) |
|  | 9. Pacific | 15,219 (13.1) | 11,358 (63.0) | 298 (6.1) | 909 (17.3) | 302 (1.2) | 1,253 (9.5) | 1,099 (2.8) |
| 2020 | I. New England | 21,330 (5.1) | 19,423 (83.6) | 459 (4.9) | 711 (6.5) | 125 (0.5) | 173 (2.7) | 439 (1.7) |
|  | 2. Middle Atlantic | 12,361 (12.7) | 9,637 (71.1) | 1,082 (11.7) | 964 (11.1) | 61 (0.4) | 271 (4.4) | 346 (1.3) |
|  | 3. East North Central | 16,354 (14.7) | 14,276 (81.3) | 1,192 (10.9) | 294 (4.2) | 159 (0.7) | 111 (1.9) | 322 (1.1) |
|  | 4. West North Central | 30,057 (6.9) | 27,352 (88.3) | 830 (5.7) | 648 (2.5) | 706 (1.2) | 139 (1.2) | 382 (1.1) |
|  | 5. South Atlantic | 28,412 (21.2) | 21,313 (67.6) | 4,894 (18.4) | 1,002 (9.5) | 256 (0.9) | 305 (2.0) | 642 (1.6) |
|  | 6. East South Central | 8,217 (5.8) | 6,032 (78.6) | 1,861 (17.4) | 69 (1.4) | 70 (0.8) | 48 (0.6) | 137 (1.2) |
|  | 7. West South Central | 9,844 (11.2) | 6,891 (58.6) | 1,076 (14.7) | 1,265 (22.8) | 223 (1.1) | 56 (1.6) | 333 (1.3) |
|  | 8. Mountain | 23,163 (7.3) | 19,033 (73.1) | 312 (3.6) | 2,383 (17.0) | 785 (2.6) | 185 (2.1) | 465 (1.6) |
|  | 9. Pacific | 14,218 (14.9) | 10,350 (57.0) | 239 (5.9) | 854 (22.0) | 355 (1.3) | 1,532 (10.9) | 888 (3.0) |

Abbreviations: AI/AN, American Indian/Alaskan Natives; API, non-Hispanic Asian and Pacific Islander or Native Hawaiian

*US Census Bureau Divisions with states in each division: Division 1: New England (Connecticut, Maine, Massachusetts, New Hampshire, Rhode Island, Vermont), Division 2: Middle Atlantic (New Jersey, New York, Pennsylvania), Division 3: East North Central (Indiana, Illinois, Michigan, Ohio, Wisconsin), Division 4: West North Central (Iowa, Kansas, Minnesota, Missouri, Nebraska, North Dakota, South Dakota), Division 5: South Atlantic (Delaware, District of Columbia, Florida, Georgia, Maryland, North Carolina, South Carolina, Virginia, West Virginia), Division 6: East South Central (Alabama, Kentucky, Mississippi, Tennessee), Division 7: West South central (Arkansas, Louisiana, Oklahoma, Texas), Division 8: Mountain (Arizona, Colorado, Idaho, New Mexico, Montana, Utah, Nevada, Wyoming), and Division 9: Pacific (Alaska, California, Hawaii, Oregon, Washington).

**Appendix Table 4.** Weighted percentage of up-to-date colorectal cancer screening rates among respondents aged 50-75 years, across 50 United States and Washington DC, Behavioral Risk Factor Surveillance System (BRFSS) 2014 and 2020

| **States** | **BRFSS 2014**  **Weighted %** | **BRFSS 2020**  **Weighted %** |
| --- | --- | --- |
| Alabama | 64.6 | 76.8 |
| Alaska | 60.0 | 70.0 |
| Arizona | 63.9 | 69.4 |
| Arkansas | 60.8 | 71.5 |
| California | 67.0 | 62.3 |
| Colorado | 67.3 | 73.9 |
| Connecticut | 73.4 | 77.5 |
| Delaware | 71.8 | 76.9 |
| District of Columbia | 67.8 | 78.6 |
| Florida | 67.1 | 75.9 |
| Georgia | 66.3 | 71.1 |
| Hawaii | 69.6 | 77.1 |
| Idaho | 61.0 | 68.1 |
| Illinois | 61.7 | 70.1 |
| Indiana | 61.6 | 71.3 |
| Iowa | 67.4 | 74.5 |
| Kansas | 64.7 | 69.7 |
| Kentucky | 67.2 | 74.5 |
| Louisiana | 64.1 | 73.0 |
| Maine | 75.4 | 81.2 |
| Maryland | 70.5 | 76.8 |
| Massachusetts | 76.5 | 80.8 |
| Michigan | 71.2 | 78.1 |
| Minnesota | 71.6 | 77.1 |
| Mississippi | 60.2 | 71.0 |
| Missouri | 61.9 | 71.7 |
| Montana | 62.5 | 69.5 |
| Nebraska | 64.0 | 72.3 |
| Nevada | 59.5 | 70.8 |
| New Hampshire | 73.9 | 78.3 |
| New Jersey | 65.1 | 71.5 |
| New Mexico | 61.4 | 68.7 |
| New York | 68.1 | 76.6 |
| North Carolina | 71.1 | 75.3 |
| North Dakota | 62.1 | 71.8 |
| Ohio | 65.2 | 74.5 |
| Oklahoma | 58.4 | 65.9 |

**Appendix Table 4 (continued).** Weighted percentage of up-to-date colorectal cancer screening rates among respondents aged 50-75 years, across 50 United States and Washington DC, Behavioral Risk Factor Surveillance System (BRFSS) 2014 and 2020

| **States** | **BRFSS 2014**  **Weighted %** | **BRFSS 2020**  **Weighted %** |
| --- | --- | --- |
| Oregon | 67.2 | 74.5 |
| Pennsylvania | 67.2 | 78.0 |
| Rhode Island | 75.2 | 80.9 |
| South Carolina | 67.5 | 76.2 |
| South Dakota | 66.9 | 76.2 |
| Tennessee | 65.3 | 75.5 |
| Texas | 60.7 | 66.9 |
| Utah | 70.2 | 74.6 |
| Vermont | 70.7 | 77.5 |
| Virginia | 69.2 | 75.6 |
| Washington | 69.6 | 74.4 |
| West Virginia | 64.3 | 71.2 |
| Wisconsin | 73.3 | 67.8 |
| Wyoming | 57.1 | 63.4 |

**Appendix Table 5.** Weighted percentage of up-to-date colorectal cancer screening rates among respondents aged 50-75 years, overall and by race/ethnicities across US Census Bureau Divisions, BRFSS 2014 and 2020

| **US Census Bureau Divisions*** | **Survey years**  **% Change** | **Overall** | **Non-Hispanic White** | **Non-Hispanic Black** | **Hispanic** | **Non-Hispanic AI/AN** | **Non-Hispanic Asian/PI** | **Non-Hispanic Other** |
| --- | --- | --- | --- | --- | --- | --- | --- | --- |
| **Division 1**  New England | 2014 | 75.01 | 76.12 | 69.44 | 65.50 | 57.64 | 66.28 | 75.18 |
|  | 2020 | 79.66 | 81.44 | 70.05 | 74.85 | 53.20 | 61.88 | 74.66 |
|  | *Absolute Change %* | *4.65* | *5.32* | *0.61* | *9.35* | *-4.44* | *-4.40* | *-0.52* |
|  | *Relative Change %* | *6.20* | *6.99* | *0.88* | *14.27* | *-7.70* | *-6.64* | *-0.69* |
|  | *AOR (95% CI)* | *1.27 (1.17-1.38)* | *1.30 (1.20-1.42)* | *1.03 (0.65-1.66)* | *1.49 (1.02-2.18)* | *1.21 (0.56-2.60)* | *0.89 (0.43-1.87)* | *0.95 (0.53-1.72)* |
|  | *P-value* | ***<0.001*** | ***<0.001*** | *0.884* | *0.041* | *0.633* | *0.761* | *0.868* |
| **Division 2**  Middle Atlantic | 2014 | 67.15 | 68.65 | 66.44 | 63.08 | 46.57 | 56.87 | 61.86 |
|  | 2020 | 75.98 | 76.62 | 79.03 | 72.33 | 73.26 | 69.18 | 68.68 |
|  | *Absolute Change %* | *8.83* | *7.97* | *12.59* | *9.25* | *26.69* | *12.31* | *6.82* |
|  | *Relative Change %* | *13.15* | *11.61* | *18.95* | *14.66* | *57.31* | *21.65* | *11.02* |
|  | *AOR (95% CI)* | *1.49 (1.37-1.62)* | *1.42 (1.29-1.55)* | *1.81 (1.39-2.35)* | *1.53 (1.13-2.07)* | *3.73 (1.12-12.47)* | *1.67 (1.01-2.76)* | *1.27 (0.65-2.50)* |
|  | *P-value* | ***<0.001*** | ***<0.001*** | ***<0.001*** | *0.006* | ***0.032*** | ***0.047*** | *0.486* |
| **Division 3**  East North Central | 2014 | 66.10 | 66.96 | 66.47 | 51.35 | 62.99 | 56.08 | 53.99 |
|  | 2020 | 72.76 | 73.77 | 72.05 | 65.26 | 66.59 | 58.14 | 61.45 |
|  | *Absolute Change %* | *6.66* | *6.81* | *5.58* | *13.91* | *3.60* | *2.06* | *7.46* |
|  | *Relative Change %* | *10.08* | *10.17* | *8.39* | *27.09* | *5.72* | *3.67* | *13.82* |
|  | *AOR (95% CI)* | *1.31 (1.22-1.41)* | *1.29 (1.20-1.39)* | *1.32 (1.00-1.73)* | *1.75 (1.00-3.06)* | *1.20 (0.47-3.09)* | *1.14 (0.55-2.36)* | *1.34 (0.82-2.21)* |
|  | *P-value* | ***<0.001*** | ***<0.001*** | ***0.046*** | *0.049* | *0.699* | *0.724* | *0.247* |
| **Division 4**  West North Central | 2014 | 66.02 | 66.94 | 63.50 | 42.45 | 60.47 | 52.47 | 58.37 |
|  | 2020 | 73.52 | 74.19 | 74.76 | 56.63 | 59.91 | 73.39 | 67.13 |
|  | *Absolute Change %* | *7.50* | *7.25* | *11.26* | *14.18* | ***-0.56*** | *20.92* | *8.76* |
|  | *Relative Change %* | *11.36* | *10.83* | *17.73* | *33.40* | ***-0.93*** | *39.87* | *15.01* |
|  | *AOR (95% CI)* | *1.38 (1.30-1.46)* | *1.34 (1.27-1.42)* | *1.69 (1.24-2.32)* | *1.74 (1.24-2.45)* | *0.94 (0.58-1.52)* | *2.56 (1.20-5.50)* | *1.52 (0.94-2.48)* |
|  | *P-value* | ***<0.001*** | ***<0.001*** | ***0.001*** | *0.001* | *0.802* | ***0.015*** | *0.092* |

**Appendix Table 5 (Continued).** Weighted percentage of up-to-date colorectal cancer screening rates among respondents aged 50-75 years, overall and by race/ethnicities across US Census Bureau Divisions, Behavioral Risk Factor Surveillance System 2014 and 2020

| **US Census Bureau Divisions*** | **Survey years**  **% Change** | **Overall** | **Non-Hispanic White** | **Non-Hispanic Black** | **Hispanic** | **Non-Hispanic AI/AN** | **Non-Hispanic Asian/PI** | **Non-Hispanic Other** |
| --- | --- | --- | --- | --- | --- | --- | --- | --- |
| **Division 5**  South Atlantic | 2014 | 68.22 | 70.09 | 68.09 | 53.59 | 60.81 | 59.36 | 66.36 |
|  | 2020 | 75.08 | 75.64 | 78.25 | 67.22 | 66.67 | 73.46 | 68.46 |
|  | *Absolute Change %* | *6.86* | *5.55* | *10.16* | *13.63* | *5.86* | *14.10* | *2.10* |
|  | *Relative Change %* | *10.06* | *7.92* | *14.92* | *25.43* | *9.64* | *23.75* | *3.16* |
|  | *AOR (95% CI)* | *1.36 (1.27-1.46)* | *1.25 (1.16-1.35)* | *1.60 (1.35-1.91)* | *1.80 (1.25-2.60)* | *1.37 (0.70-2.69)* | *1.75 (1.05-2.91)* | *1.12 (0.72-1.74)* |
|  | *P-value* | ***<0.001*** | ***<0.001*** | ***<0.001*** | ***0.001*** | *0.358* | ***0.030*** | *0.615* |
| **Division 6**  East South central | 2014 | 64.84 | 65.47 | 63.28 | 60.20 | 59.50 | 31.67 | 60.63 |
|  | 2020 | 74.95 | 75.50 | 75.02 | 60.09 | 51.09 | 71.67 | 72.84 |
|  | *Absolute Change %* | *10.11* | *10.03* | *11.74* | *-0.11* | *-8.41* | *40.00* | *12.21* |
|  | *Relative Change %* | *15.59* | *15.32* | *18.55* | *-0.18* | *-14.13* | *126.30* | *20.14* |
|  | *AOR (95% CI)* | *1.58 (1.44-1.73)* | *1.57 (1.42-1.74)* | *1.68 (1.33-2.12)* | *0.85 (0.31-2.33)* | *0.74 (0.29-1.92)* | *7.82 (1.98-30.82)* | *1.65 (0.78-3.48)* |
|  | *P-value* | ***<0.001*** | ***<0.001*** | ***<0.001*** | *0.748* | *0.538* | ***0.003*** | *0.189* |
| **Division 7**  West South Central | 2014 | 60.93 | 64.67 | 69.58 | 43.07 | 63.52 | 44.51 | 60.77 |
|  | 2020 | 67.79 | 72.00 | 71.97 | 56.82 | 55.87 | 53.08 | 51.06 |
|  | *Absolute Change %* | *6.86* | *7.33* | *2.39* | *13.75* | *-7.65* | *8.57* | *-9.71* |
|  | *Relative Change %* | *11.26* | *11.33* | *3.43* | *31.92* | *-12.04* | *19.25* | *-15.98* |
|  | *AOR (95% CI)* | *1.35 (1.19-1.53)* | 1.33 (1.16-1.52) | *1.03 (0.70-1.51)* | *1.75 (1.26-2.42)* | *0.76 (0.45-1.29)* | *1.38 (0.46-4.14)* | *0.69 (0.39-1.22)* |
|  | *P-value* | ***<0.001*** | **<0.001** | *0.881* | ***0.001*** | *0.311* | *0.562* | *0.205* |
| **Division 8**  Mountain | 2014 | 64.00 | 66.49 | 69.67 | 52.40 | 48.99 | 58.99 | 60.89 |
|  | 2020 | 70.78 | 73.12 | 68.47 | 63.57 | 60.69 | 70.88 | 62.24 |
|  | *Absolute Change %* | *6.78* | *6.63* | ***-1.2*** | *11.17* | *11.70* | *11.89* | *1.35* |
|  | *Relative Change %* | *10.59* | *9.97* | ***-1.72*** | *21.32* | *23.88* | *20.16* | *2.22* |
|  | *AOR (95% CI)* | *1.32 (1.24-1.40)* | 1.28 (1.20-1.36) | *0.85 (0.52-1.40)* | *1.56 (1.31-1.86)* | *1.51 (1.06-2.15)* | *1.87 (0.97-3.62)* | *0.95 (0.62-1.46)* |
|  | *P-value* | ***<0.001*** | **<0.001** | *0.531* | ***<0.001*** | ***0.021*** | *0.063* | *0.826* |

**Appendix Table 5 (Continued).** Weighted percentage of up-to-date colorectal cancer screening rates among respondents aged 50-75 years, overall and by race/ethnicities across US Census Bureau Divisions, Behavioral Risk Factor Surveillance System 2014 and 2020

| **US Census Bureau Divisions*** | **Survey years**  **% Change** | **Overall** | **Non-Hispanic White** | **Non-Hispanic Black** | **Hispanic** | **Non-Hispanic AI/AN** | **Non-Hispanic Asian/PI** | **Non-Hispanic Other** |
| --- | --- | --- | --- | --- | --- | --- | --- | --- |
| **Division 9**  Pacific | 2014 | 67.43 | 71.38 | 77.79 | 49.39 | 51.89 | 69.60 | 67.04 |
|  | 2020 | 65.95 | 70.69 | 63.05 | 59.18 | 66.35 | 56.74 | 64.72 |
|  | *Absolute Change %* | *-1.48* | *-0.69* | ***-14.74*** | *9.79* | *14.46* | ***-12.86*** | *-2.32* |
|  | *Relative Change %* | *-2.19* | *-0.97* | ***-18.95*** | *19.82* | *27.87* | ***-18.48*** | *-3.46* |
|  | *AOR (95% CI)* | *0.93 (0.81-1.06)* | *0.90 (0.78-1.04)* | ***0.47 (0.23-0.96)*** | *1.46 (1.04-2.06)* | *1.84 (0.67-5.06)* | ***0.54 (0.31-0.95)*** | *0.88 (0.50-1.55)* |
|  | *P-value* | *0.274* | *0.161* | ***0.039*** | ***0.028*** | *0.235* | ***0.034*** | *0.661* |

Relative change percent was calculated by dividing the difference in screening rates (2020 minus 2014) by the 2014 rate and then multiplied by 100. Adjusted odds ratio (AOR) and *p*-values are from adjusted logistic regression models of change in screening rates in 2020 vs. 2014. (Overall model adjusted for age, sex, and race/ethnicity, while racial ethnic subgroup models adjusted for age and sex only)

*US Census Bureau Divisions with states in each division: Division 1: New England (Connecticut, Maine, Massachusetts, New Hampshire, Rhode Island, Vermont), Division 2: Middle Atlantic (New Jersey, New York, Pennsylvania), Division 3: East North Central (Indiana, Illinois, Michigan, Ohio, Wisconsin), Division 4: West North Central (Iowa, Kansas, Minnesota, Missouri, Nebraska, North Dakota, South Dakota), Division 5: South Atlantic (Delaware, District of Columbia, Florida, Georgia, Maryland, North Carolina, South Carolina, Virginia, West Virginia), Division 6: East South Central (Alabama, Kentucky, Mississippi, Tennessee), Division 7: West South central (Arkansas, Louisiana, Oklahoma, Texas), Division 8: Mountain (Arizona, Colorado, Idaho, New Mexico, Montana, Utah, Nevada, Wyoming), and Division 9: Pacific (Alaska, California, Hawaii, Oregon, Washington).
